# Supplementary material for: An updated compendium of Caenorhabditis elegans RNA-binding proteins and their regulation
Source: G3 (Bethesda). 2025 Jul 14;15(9):jkaf156. doi: 10.1093/g3journal/jkaf156 (PMC12405884; doi:10.1093/g3journal/jkaf156)
Supplement: jkaf156_Supplementary_Data [file jkaf156_supplementary_data.zip › Supplemental_Figure_S1_G3-2025-405833.pdf]

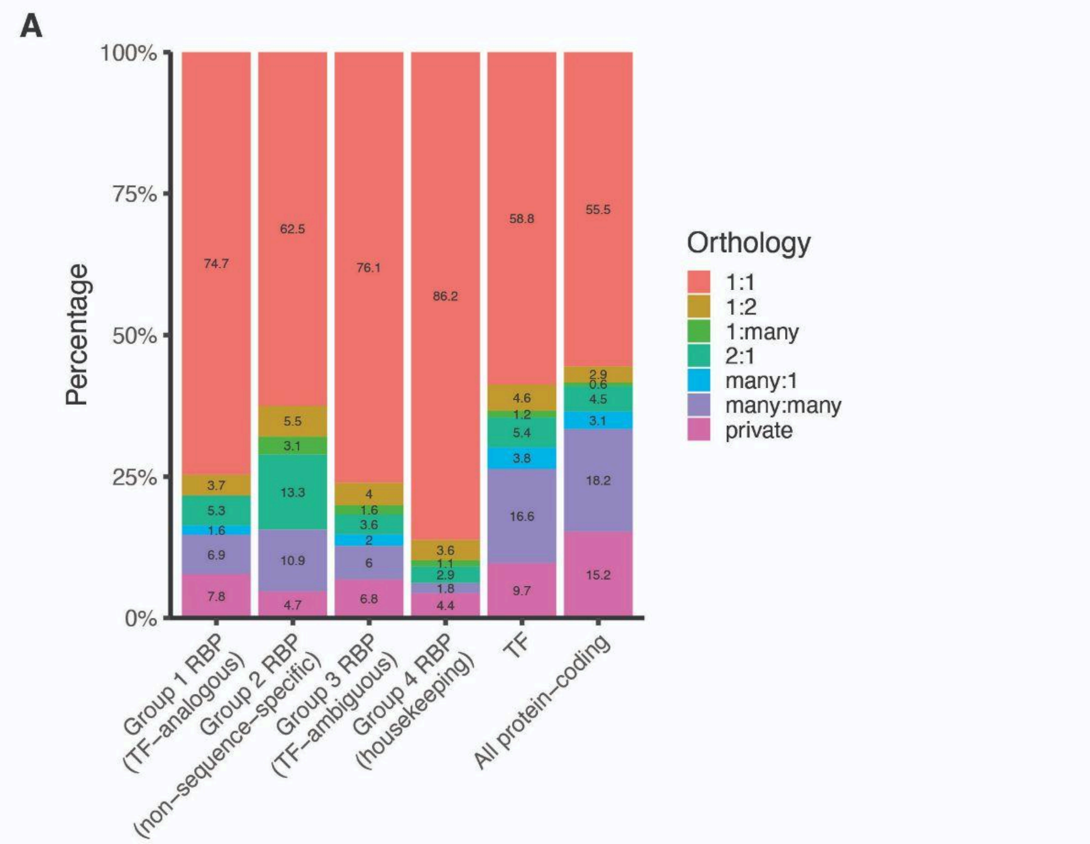

B

Chi-Square Test p-values, compared to all protein-coding genes

	1:1	1:2	1:Many	2:1	Many:1	Many:Many	Private
Group 1 RBPs	1.154e-07	0.5185	0.2061	0.6485	0.1677	2.1e-06	0.0005895
Group 2 RBPs	0.6293	0.1255	0.0007574	1.231e-05	0.03597	0.01329	0.0003432
Group 3 RBPs	1.322e-09	0.3254	0.05837	0.4306	0.2969	2.936e-07	0.0001216
Group 4 RBPs	< 2.2e-16	0.4659	0.3374	0.1867	0.003017	1.453e-12	4.12e-07
TF	0.1866	0.006899	0.08254	0.3319	0.321	0.2323	3.115e-05

Supplemental Figure S1. The percentage of RBP and TF genes with various orthology groups to *C. briggsae* genes differs compared to that of all protein-coding genes. (A) The percentage of genes in each gene category with a given orthology relationship to *C. briggsae* genes. Orthology data taken from (Large et al. 2024). (B) A Chi-Square Test was used to determine statistically significant differences in each group compared to all protein-coding genes. P-values of all Chi-Square Tests are shown in the table.
